# Monolithic Multicolor Emissions of InGaN-Based Hybrid Light-Emitting Diodes Using CsPbBr_3_ Green Quantum Dots

**DOI:** 10.3390/ma16031290

**Published:** 2023-02-02

**Authors:** Jae-Hyeok Oh, Seung-Beom Cho, Il-Kyu Park, Sung-Nam Lee

**Affiliations:** 1 Department of IT & Semiconductor Convergence Engineering, Tech University of Korea, Siheung 15073, Republic of Korea; 2 Department of Materials and Science Engineering, Seoul National University of Science & Technology, Seoul 01811, Republic of Korea; 3 Department of Nano & Semiconductor Engineering, Tech University of Korea, Siheung 15073, Republic of Korea

**Keywords:** multicolor emission, InGaN, hybrid light-emitting diode, CsPbBr_3_, quantum dot, light conversion, injection current

## Abstract

To address the increasing demand for multicolor light-emitting diodes (LEDs), a monolithic multicolor LED with a simple process and high reliability is desirable. In this study, organic–inorganic hybrid LEDs with violet and green wavelengths were fabricated by depositing CsPbBr_3_ perovskite green quantum dots (QDs) as the light-converting material on InGaN-based violet LEDs. As the injection current was increased, the total electroluminescence (EL) intensities of the hybrid LEDs increased, whereas the light-converted green emission efficiency of the CsPbBr_3_ QDs decreased. The maximum green-to-violet EL spectral intensity ratio of the hybrid LEDs with CsPbBr_3_ QDs was achieved with the injection current of <10 mA. Moreover, the EL spectral ratio of the green-to-violet emission decreased at an injection current of 100 mA. The light-conversion intensity of the CsPbBr_3_ QDs decreased linearly as the junction temperature of the hybrid LEDs was increased with increasing injection current, similar to the temperature-dependent photoluminescence degradation of CsPbBr_3_ QDs. In addition, the junction temperature of the hybrid LED was minimized by pulse injection to suppress the thermal degradation of QDs and increase the light conversion efficiency to green emission. Therefore, the overall emission spectrum color coordinates of the hybrid LEDs exhibited a red shift from violet to blue in the low-current region and a blue shift toward violet as the green emission of the QDs was decreased above 10 mA.

## 1. Introduction

The development of multicolor light-emitting diodes (LEDs) is promising for multifunctional lighting sources, such as display, biomedical, agricultural, and cosmetic applications [1,2,3]. To fabricate a multifunctional lighting source, two or three individual LEDs are integrated to realize dichromatic or trichromatic multicolor LEDs [1,4]. However, as multicolor LEDs use two kinds of LED and additional integration technology, the device reliability is lower than that of monolithic multicolor LEDs, and the fabrication process is more complicated [5,6]. Therefore, a monolithic multicolor LED with a simple process and high reliability for various applications is desirable.

Recently, III-nitride semiconductors have garnered considerable attention as the most common visible light-emitting materials that can achieve stable wavelengths by controlling the compositions of ternary or quaternary compound semiconductors [7,8]. In particular, InGaN ternary semiconductors have been widely used as blue and green light sources by controlling the In composition of the InGaN active layer [7]. Therefore, InGaN-based monolithic LEDs have been fabricated by the growth of dual-emission InGaN active layers, such as self-organized InGaN quantum dots (QDs) and blue/yellow dual InGaN quantum wells (QWs), using different In compositions and well thicknesses [9,10,11]. However, high-efficiency green and blue emissions cannot be easily simultaneously achieved from InGaN-based LEDs because of the In segregation and large lattice mismatch [12,13,14].

To address the limitations of InGaN-based monolithic LEDs, high-efficiency InGaN-based LEDs with light-conversion materials, such as CuInS_2_, yttrium aluminum garnet (YAG), and silicate-based phosphors, have garnered attention for the fabrication process [15,16,17]. CuInS_2_ exhibits a wide half-width, making it suitable as a light-converting device for lighting sources, whereas YAG- and silicate-based phosphors have low color purity and reliability, which are not suitable for multiwavelength LEDs [15,16,17]. CdSe–ZnS nanocrystals, which adsorb high-energy blue emissions generated from InGaN-based LEDs, have been reported to convert low-energy green or yellow emissions [18].

Recently, perovskite QDs have been studied to improve light-conversion efficiency due to their Cd-free properties [19,20]. In particular, CsPbBr_3_ perovskite QDs are superior to conventional QD materials because of their high light absorption coefficient and stability [19,20,21]. In addition, CsPbBr_3_ QDs have a pure green photoluminescence (PL) emission of approximately 520 nm with a narrower bandwidth and higher quantum efficiency of ~90% [21]. Thus, CsPbBr_3_ QDs are considered as candidates for realizing converted green emissions using high-energy emission LEDs because of their absorption and emission properties. In particular, hybrid LEDs combining green CsPbBr_3_ and blue-violet InGaN-based LEDs are expected to achieve a multiwavelength LED that can control a wide range of emission wavelengths from blue-violet to green by controlling the conversion efficiency.

In this study, organic–inorganic hybrid LEDs with a mixed violet and green wavelength range were fabricated by depositing CsPbBr_3_ perovskite green QDs as the light-converting material on InGaN-based violet LEDs. The light-conversion performance of the materials was tested under different injection currents and temperatures.

## 2. Materials and Methods

CsPbBr_3_ QDs were synthesized using a modified hot injection method [22,23]. Briefly, 325 mg Cs_2_CO_3_ dissolved in a mixture of 1 mL oleic acid and 3 mL 1-octadecene was vacuum-dried in a three-neck flask at 100 °C for 30 min to synthesize the Cs-oleate solution. In another flask, 138 mg PbBr_2_ was dissolved in a mixture of 1 mL oleic acid, 1 mL oleylamine, and 10 mL 1-octadecene. The solution was vacuum dried in a three-neck flask at 120 °C for 30 min and heated to 180 °C. Subsequently, 1 mL Cs-oleate solution was injected into the reaction flask. After 7 s, the reaction flask was dipped into an ice-water bath to stop the reaction. To remove the unreacted white solid and excess ligand residues, the crude solution was centrifuged at 10,000 rpm for 3 min. The QD inks were washed with ethyl acetate with a volume ratio of 3:1, followed by re-precipitation to produce a pure colloidal QD solution. Then, the QDs were re-dispersed into an *n*-octane solution for further use.

The InGaN-based LED epitaxial structure was grown on a c-plane (0001) sapphire substrate using metal–organic chemical deposition. Trimethylgallium, trimethylindium, trimethylaluminum, and ammonia were used as Ga, In, Al, and N precursors, respectively. The InGaN-based violet LEDs consisted of 4.0 µm-thick Si-doped n-GaN, five-period In_0.1_Ga_0.9_N/GaN QWs, 20 nm-thick p-AlGaN electron-blocking layer, and 0.1 µm-thick Mg-doped p-GaN. The growth temperatures of n-type and p-type GaN films are 1030 and 900 °C, respectively. As the active layer, InGaN/GaN 5-QW structures composed of 3.0 nm-thick In_0.1_Ga_0.9_N wells and 10.0 nm-thick GaN barriers were grown at 800 °C. Subsequently, LED chips with lateral electrode structures were fabricated using a conventional mesa LED process. After depositing the Ni/Au p-electrode on the LEDs, CsPbBr_3_ QDs were deposited on the InGaN-based violet LEDs using a spin-coating process. For the spin-coating method, 50 µL CsPbBr_3_ was added at 2000 rpm for 60 s, and then the n-hexane solvent was removed at 70 °C for 20 min in a nitrogen atmosphere.

The microstructure of the CsPbBr_3_ QDs was observed using a transmission electron microscope (TEM, JEOL-2010, Tokyo, Japan) at 200 kV. To measure the TEM, 10 μL of CsPbBr_3_ QD solution was dropped onto the copper grid. They were dried in a vacuum oven at 60 °C for 30 min to remove the organic substances, which could interrupt a clear TEM image. The surface structures of the CsPbBr_3_ QDs on glass, InGaN-based LEDs and hybrid LEDs were measured using noncontact mode atomic force microscopy (AFM). Optical absorption and transmittance of the CsPbBr_3_ QDs on glass and InGaN-based LEDs were obtained using UV-visible spectroscopy. In addition, the optical emission properties of the CsPbBr_3_ QDs, InGaN-based violet LEDs, and hybrid LEDs were evaluated by room-temperature PL measurements using a 266 nm laser. To observe the effects of QD deposition on the current, voltage, and emission intensity of the hybrid LEDs, light output power (L)–current (I)–voltage (V) measurements were carried out using an HP4155A parameter analyzer with a 918D-UV-OD3R Newport photodetector capable of current injection and photocurrent measurements. In addition, electroluminescence (EL) spectra of LEDs were obtained by USB 4000 fiber optic spectrometer. The junction temperature of the hybrid LEDs was measured using the forward voltage method [24]. In addition, temperature-dependent PL measurements at 25–100 °C were performed using a hot chuck and 405 nm laser as the excitation source to observe the optical and thermal damage of the CsPbBr_3_ QDs.

## 3. Results and Discussion

**Figure 1:**
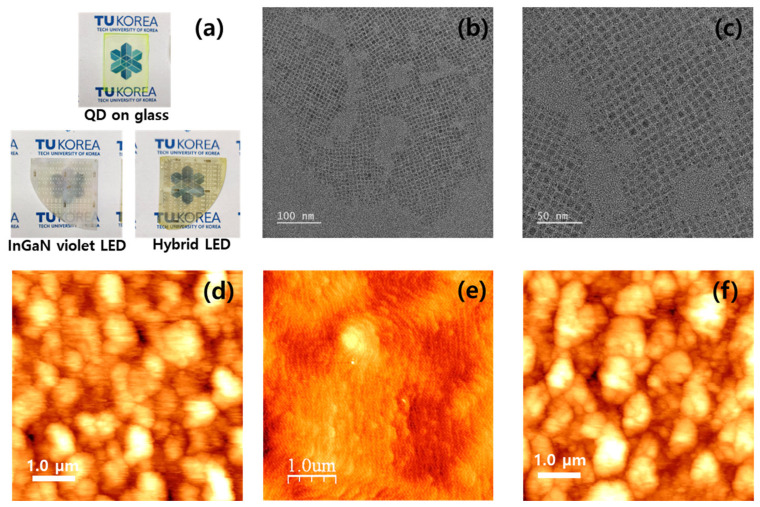
(**a**) Images of spin-coated CsPbBr_3_ on glass, conventional InGaN-based violet LEDs, and hybrid LED with CsPbBr_3_ QDs. (**b**,**c**) TEM images of the CsPbBr_3_ QDs. Surface morphologies of (**d**) spin-coated CsPbBr_3_ on glass, (**e**) InGaN-based violet LED, and (**f**) hybrid LED with CsPbBr_3_ QDs obtained by AFM.

Figure 1a shows images of the CsPbBr_3_ QDs on a glass substrate, InGaN-based violet LEDs, and hybrid InGaN-based multicolor LEDs with CsPbBr_3_ QDs. Detailed 3-dimensional schematic diagrams of QDs on glass, InGaN-based violet LED, and hybrid LED structures are described in Figure S1. CsPbBr_3_ on glass exhibits excellent transparency and a light green color. The hybrid InGaN-based LEDs have a darker yellow color than that of conventional InGaN-based violet LEDs, which was obtained by depositing CsPbBr_3_ QDs. This indicates that CsPbBr_3_ QDs can be uniformly coated on the glass substrates and InGaN-based LEDs by spin coating. Figure 1b,c show the TEM images of the synthesized all-inorganic CsPbBr_3_ QDs, which exhibited a uniform cubic shape. The black dot spots shown in higher-resolution TEM image has been known as metallic Pb particle formed by electron beam exposure [25]. The CsPbBr_3_ QDs have an average size of approximately 8.7 nm with a uniform size distribution. As the size of the QDs was slightly larger than the bulk exciton Bohr radius of 7 nm, the PL spectra did not show a blue shift due to the quantum confinement effect [26]. In addition, the CsPbBr_3_ QDs were well dispersed in the *n*-octane solution without agglomeration. This indicates that the ligand functional groups of the QDs helped stabilize the colloidal stability, thereby allowing them to be spin-coated on other surfaces, such as sapphire or GaN LED chips.

From the AFM analysis, the average surface grain size and root mean square roughness (RMS) of the CsPbBr_3_ QDs on the glass substrate was measured to be approximately 50 and 23 nm, respectively, as shown in Figure 1d. This indicates that spin coating technology is a useful deposition method that can form CsPbBr_3_ QD-coated thin films with a uniform smooth surface. Figure 1e shows the AFM surface morphology of the GaN-based violet LED with a smooth step-like and hillock surface structure, which is a typical surface morphology of GaN films [27]. This suggests the smooth surface structure of the GaN-based LEDs with an RMS surface roughness of 0.6 nm. After coating the CsPbBr_3_ QDs on the InGaN-based LEDs, the surface structure of the hybrid LEDs was significantly changed from a step-like surface to a granular spherical surface, as shown in Figure 1f. The RMS roughness of the hybrid InGaN-based LED coated with CsPbBr_3_ QDs was 26 nm, which is almost the same as that of the CsPbBr_3_ QDs on glass. This suggests that CsPbBr_3_ QDs can be coated on InGaN-based LED and glass substrates.

**Figure 2:**
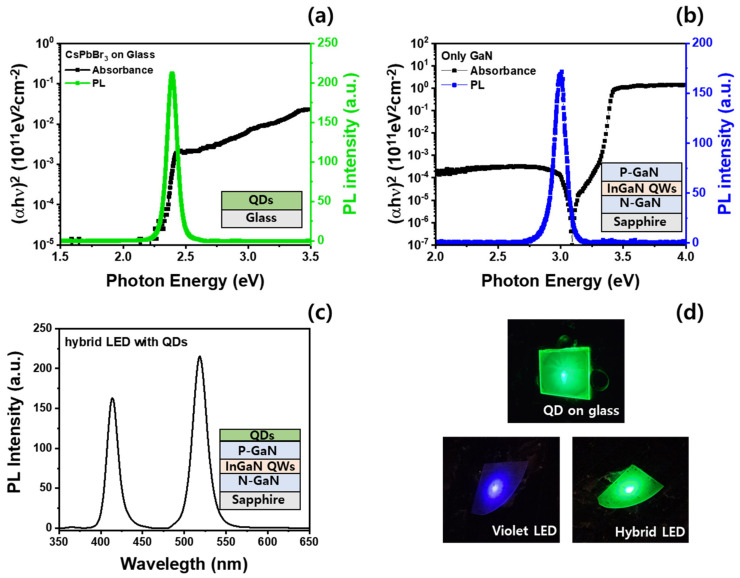
Room-temperature PL and absorption spectra of (**a**) spin-coated CsPbBr_3_ QDs on glass, (**b**) conventional InGaN-based LED, and (**c**) hybrid InGaN-based LED using CsPbBr_3_ QD; (**d**) images of the PL emissions obtained from the spin-coated CsPbBr_3_ QDs on glass, conventional InGaN-based LED, and hybrid InGaN-based LED using CsPbBr_3_ QDs.

Figure 2a shows the room-temperature PL and light absorption spectra of the CsPbBr_3_ QDs deposited on a glass substrate. The bandgap energy of the CsPbBr_3_ QDs can be estimated at the light-absorption edge to be ~2.390 eV, which is approximately equal to the emission energy (~2.391 eV) of the PL peak. The CsPbBr_3_ QDs exhibit a small Stokes shift, indicating that green QDs have excellent uniform optical properties. In addition, the full width at half maximum (FWHM) of the CsPbBr_3_ QDs PL spectrum was measured to be ~60 meV, which exhibits uniform green emission characteristics compared to the InGaN-based LED. Figure 2b shows the PL and light-absorption spectra of the InGaN-based violet LED. A sharp absorption edge was measured at 3.380 eV, corresponding to the GaN template. A significant change in the absorption coefficient corresponding to the InGaN active layer was observed at 3.095 eV. Moreover, the PL peak energy of the InGaN-based LED was approximately 3.024 eV, which exhibits a Stokes shift of approximately 71 meV from the bandgap of the InGaN active layer obtained by light absorption. This can be ascribed to the slightly non-uniform In composition or well thickness in the five-period InGaN/GaN QW structure. Figure 2c shows the PL spectra of the hybrid LED with CsPbBr_3_ QDs as the light converter. Two strong peaks at 410 and 517 nm were observed for the hybrid InGaN-based LED with CsPbBr_3_ QDs. From Figure 2a,b, emissions at 410 and 518 nm were noted from the violet InGaN active layer and CsPbBr_3_ green QDs, respectively. The PL intensity of the CsPbBr_3_ green QDs was 20% higher than that of the InGaN-based violet LEDs. However, no evidence pertains to the superior optical quality of the CsPbBr_3_ QDs to that of the InGaN-based LEDs. As the excitation light source first excited the upper green QDs on the LED and passed through the p-GaN upper layer to excite the InGaN active layer, the violet emission of the InGaN active layer is lower than the green QD emission. However, the CsPbBr_3_ QDs were coated sufficiently well to emit green emissions by exciting a high-energy light source on InGaN-based LED using a spin-coating process.

**Figure 3:**
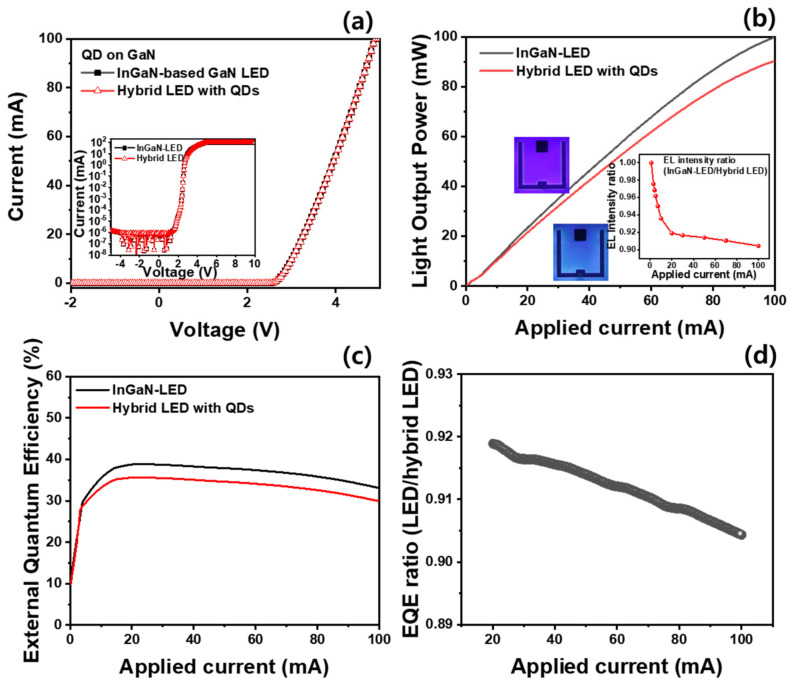
L–I–V characteristics of the InGaN-based and hybrid LEDs: (**a**) I–V curves, (**b**) light output power versus injection current, and (**c**) EQE of InGaN-based violet LED and hybrid LED with CsPbBr_3_ QDs; (**d**) EQE ratio of InGaN-based violet LEDs to hybrid LEDs with CsPbBr_3_ QDs as a function of the injection current.

Figure 3a shows the I–V characteristics of the conventional InGaN-based violet LED and hybrid LEDs with CsPbBr_3_ QDs. The threshold and operation voltages for both LEDs are 2.7 and 3.2 V, respectively. In addition, the log-scaled I–V curves of the two LEDs have identical forward and reverse electrical characteristics, as shown in the inset of Figure 3a. This indicates that the spin-coating process of the CsPbBr_3_ QDs proceeded well without electrical damage to the violet LED because the CsPbBr_3_ QDs were coated after the p-type electrode deposition of the LED was formed. However, the light output powers of a conventional InGaN-based violet LED and hybrid LED coated with CsPbBr_3_ QDs are similar in the low-current region (<10 mA), whereas the light output power of the hybrid LED using CsPbBr_3_ QDs is slightly lower than that of conventional InGaN-based violet LED in the high-current region (>20 mA), as shown in Figure 3b. The difference in the light output power between the two LEDs at an injection current of 5.0 mA was less than 3.0%. However, when a current of 100 mA was injected, the light output power of the hybrid LED was 90.4% that of the InGaN-based violet LEDs, as shown in the inset of Figure 3b. The external quantum efficiencies (EQE) of the InGaN-based violet LEDs and hybrid LEDs with CsPbBr_3_ QDs showed a typical efficiency droop with maximum values of 38.8% and 35.6% at 20 mA, respectively, as shown in Figure 3c. This indicates that the EQE of the InGaN-based violet LEDs decreased by coating with CsPbBr_3_ QDs. In particular, the EQE of the InGaN-based violet LEDs was higher than that of the hybrid LEDs under an injection current of more than 10 mA because of the light conversion efficiency of the CsPbBr_3_ QDs under the excitation light (~410 nm) of the InGaN-based violet LEDs. Above the maximum EQE of 20 mA, the EQE reduction ratio of the InGaN-based violet LEDs to the hybrid LEDs was proportional to the injection current, as shown in Figure 3d. This indicates that the light conversion efficiency of the CsPbBr_3_ QDs decreased proportionally with the injection current. The optical properties of CsPbBr_3_ perovskite QDs with an ABX_3_ structure are vulnerable to moisture and heat due to oxygen diffusion and structural decomposition [28,29]. Hence, the heat generated by the InGaN-based violet LEDs under a high injection current is expected to deteriorate the light conversion efficiency of CsPbBr_3_ QDs, resulting in an EQE decrease in the hybrid LEDs.

**Figure 4:**
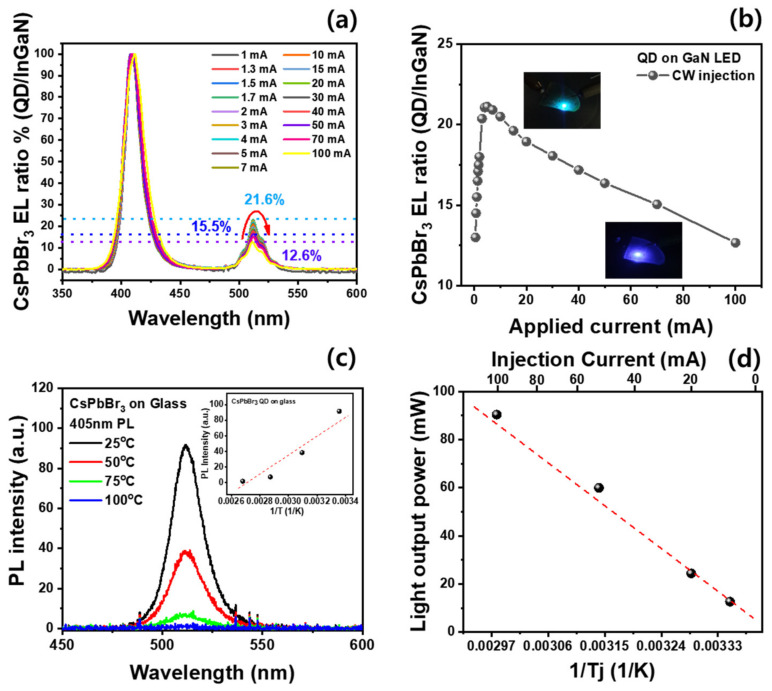
(**a**) Normalized EL spectra of the hybrid LEDs with CsPbBr_3_ QDs under different injection currents. (**b**) EL intensity ratio of the green to violet emissions in hybrid LEDs. (**c**) Temperature-dependent PL spectra of the CsPbBr_3_ QDs on glass substrate. The inset is the PL intensity of the CsPbBr_3_ QDs as a function of reciprocal temperature. (**d**) The light output power of the hybrid LEDs as a function of the reciprocal junction temperature and injection current.

To analyze the decrease in the light output power in the region with a high injection current of the hybrid LEDs, the normalized EL of the hybrid LEDs was investigated as the injection current was increased from 0.1 to 100 mA, as illustrated in Figure 4a. Two main EL peaks were noted: violet emission (410 nm) from the InGaN active region and green emission (518 nm) from CsPbBr_3_ QDs. The EL intensity of the CsPbBr_3_ green QD emission was approximately 20% that of the violet GaN-based LEDs, indicating that the light-conversion efficiency of CsPbBr_3_ QDs is still low for the violet emission of InGaN-based LEDs. As the injection current was increased to 10 mA, the EL spectrum of the CsPbBr_3_ QDs initially increased and then rapidly decreased above 10 mA. To observe the effect of the injection current on the violet-to-green emissions of the hybrid LEDs, the EL intensity ratio of the green-to-blue-violet emissions was plotted as a function of the injection current, as shown in Figure 4b. The EL intensity ratio of green to violet emission increased to 21.6% at 5.0 mA and decreased to 12.6% at 100 mA. Thus, the maximum light conversion efficiency of the CsPbBr_3_ QDs for the violet excitation emission source was noted at a low injection current of 5.0 mA, which decreased in the high-injection-current region. From the different conversion efficiencies of the CsPbBr_3_ QDs shown in the insets of Figure 4b, the hybrid LEDs using CsPbBr_3_ QDs exhibit a bluish-green emission in the low-current region and violet emission in the high-current region. As the device temperature of LEDs increases with the injection current due to an increase in the junction temperature [30], we measured the junction temperature of the hybrid LED using the forward-voltage method [24]. As the injection current was increased from 10 mA to 100 mA, the junction temperature of the hybrid LEDs increased from 26.2 °C to 65 °C, as shown in Supplementary S2. This indicates that the hybrid LEDs have good epitaxial properties with low nonradiative recombination centers, which can generate heat in the junction region. However, the light-conversion efficiency of the CsPbBr_3_ QDs may be deteriorated by thermal degradation due to the increased junction temperature of the hybrid LEDs.

To investigate the effect of thermal degradation on the optical properties of CsPbBr_3_ QDs, temperature-dependent PL analysis was performed on CsPbBr_3_ QDs at 25–100 °C, as shown in Figure 4c. The PL intensity of the CsPbBr_3_ QDs significantly decreased with increasing ambient temperature. By using the Arrhenius equation [31], the activation energy for the temperature-dependent decrease in the PL intensity was calculated to be 50.4 meV, as shown in the inset of Figure 4c. The junction temperature of the hybrid LEDs is proportional to the injection current, as shown in Supplementary S2. By using the junction temperature of the hybrid LEDs, the EL intensity was plotted as a function of the junction temperature to calculate the activation energy for the temperature-dependent EL intensity of the hybrid LEDs, as shown in Figure 4d. The activation energy of the temperature-dependent EL intensity of the hybrid LEDs was calculated to be 44.5 meV, which is similar to that of the CsPbBr_3_ QDs. Therefore, the decrease in the EL intensity of the hybrid LEDs with increasing current can be explained by the thermal degradation of the QDs, such as the oxygen diffusion and decomposition of the unstable colloidal structure on the QD surface [29], as the temperature of the hybrid LEDs increased with the junction temperature.

**Figure 5:**
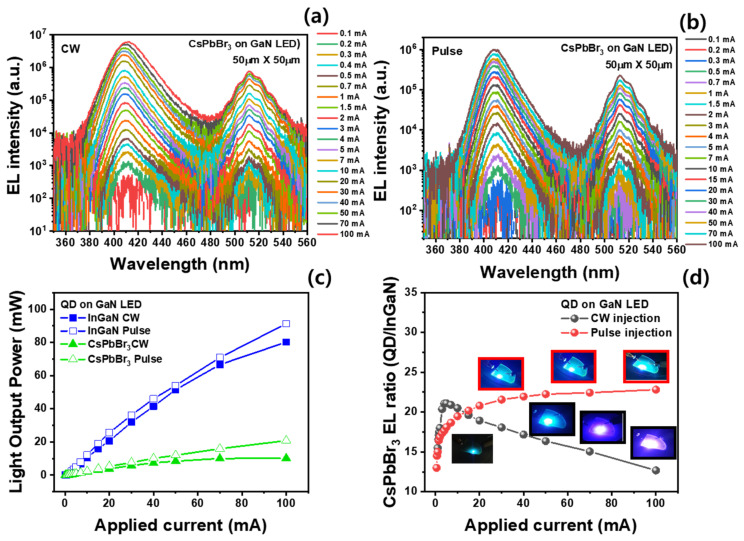
EL spectra of the hybrid LEDs with CsPbBr_3_ QDs with different (**a**) CW and (**b**) pulse injection (pulse width 5.0 μs and 1.0 % duty cycle) currents; (**c**) EL spectral intensity of the violet InGaN active layer and green CsPbBr_3_ QDs in the hybrid LEDs as a function of the CW and pulse injection currents; (**d**) EL intensity ratio of the green to violet emission in the hybrid LEDs with different CW and pulse injection currents.

Figure 5a,b show the EL spectra of the hybrid LEDs with CsPbBr_3_ QDs with different injection currents using the continuous-wave (CW) and pulse injection conditions, respectively. Under both conditions, the violet emission of the InGaN active layer is higher than that of the CsPbBr_3_ QDs. The blue-violet emission (~410 nm) exhibits a redshift with increasing continuous current injection due to the thermal heating-induced band gap shrinkage effect, whereas no redshift phenomenon was noted under the high-pulse-injection condition [32]. The FWHMs of the violet and green EL spectra obtained by the CW operation are wider than those obtained by the pulse operation, which confirms the thermal heating effect [33]. Figure 5c shows the EL integrated intensities of the violet and green emission spectra generated from the hybrid LEDs as a function of the CW and pulse injection currents. The CW EL intensity of the violet emission generated from the InGaN active layer was slightly lower than that under the pulse-injection condition. However, the CW EL intensity of the green emission from the CsPbBr_3_ QDs did not further increase above 50 mA, whereas that of the green QDs measured under pulse conditions increased almost linearly with the injection current. The EL intensity ratio of the green-to-violet emission measured under the CW and pulse injection conditions in the hybrid LEDs is shown in Figure 5d. For the CW condition, the maximum intensity ratio (21.1%) of the green to violet emission was obtained at 5.0 mA, which rapidly decreased with increasing injection current. However, the intensity ratio of the green emission to the blue-violet emission did not decrease until the pulse injection current was 100 mA. As a result, the emission intensity ratios of the green to violet LED emission measured under CW and pulse injection conditions were 12.6% and 22.8% at 100 mA, respectively. In addition, it is observed that the green emission intensity of the hybrid LED operated by pulse operation is much more stable than that of cw operation, as shown in Supplementary S3. Hence, the decrease in the emission efficiency of hybrid LEDs at high operation currents is attributed to the reduction in the light conversion efficiency of the CsPbBr_3_ green QDs due to thermal degradation such as oxygen diffusion and decomposition of unstable colloidal structure with increasing junction temperature of hybrid LEDs [29,34]. The insets of Figure 5d show the emission images of the hybrid LED wafer with different CW and pulse injection currents. The hybrid LEDs emit blue-based emissions in all pulse injection regions, and blue emissions followed by violet emission as the CW injection current was increased because of the decrease in the green emission. This indicates that the hybrid LEDs can emit various color coordinates due to the change in the emission intensity ratio of the violet to green emission under the CW and pulse injection conditions.

**Figure 6:**
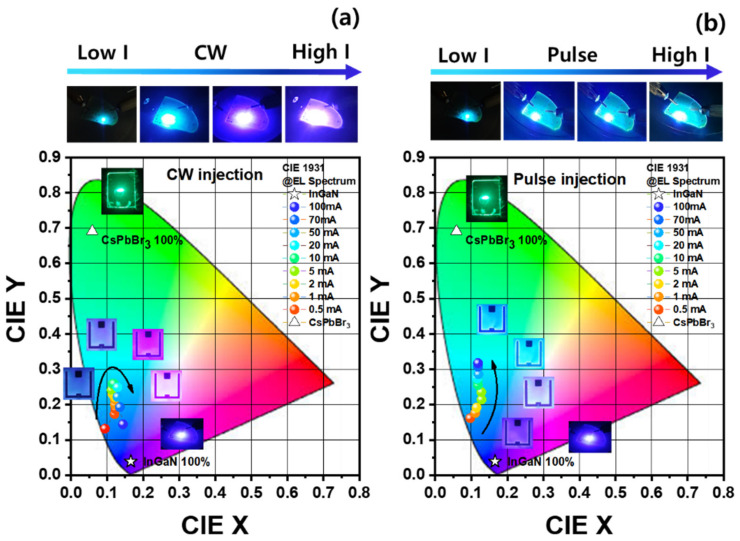
Color coordinate images of the hybrid LEDs using CsPbBr_3_ QDs under (**a**) CW and (**b**) pulse injection currents. The insets are the EL images of the hybrid LED die and wafer with increasing injection currents.

Figure 6a,b show the color coordinates of the EL spectra of the hybrid LEDs with different CW and pulse injection conditions, respectively. Under the CW injection condition, the color coordinates of (0.1206, 0.1731) of the hybrid LEDs were obtained at a low injection current of 0.5 mA. As the injection current was increased, the color coordinate of the hybrid LED shifted to the green emission point of (0.0590, 0.6914), as obtained from the CsPbBr_3_ QDs. The maximum redshifted color coordinates of (0.1185, 0.2593) were obtained at 10 mA. The color coordinates of the hybrid LEDs shifted to the violet emission region up to 100 mA due to the rapid decrease in the light-conversion efficiency of the green CsPbBr_3_ QDs, as shown in Figure 6a. However, as the pulse injection current was increased, the color coordinates of the hybrid LED shifted to the green emission region because of the lower thermal degradation of the light conversion efficiency of the CsPbBr_3_ QDs. Consequently, as the injection current was increased from 10 mA to 100 mA, the color coordinates can be further redshifted from the blue emission of (0.1160, 0.2738) to the cyan emissions of (0.1187, 0.3173), as shown in Figure 6b. Based on these results, we believe that InGaN-based hybrid LEDs with CsPbBr_3_ green QDs can achieve multicolor emissions from blue-violet to cyan emissions to minimize the thermal degradation of the light-conversion efficiency of green QDs using the pulse injection condition. In addition, the thermal and photostability of CsPbBr_3_ QDs still remain to be improved for more applications by incorporating foreign elements, passivating the surface of QDs, or encapsulating the device.

## 4. Conclusions

In summary, multicolor InGaN-based hybrid LEDs were fabricated by depositing CsPbBr_3_ green QDs as a light-conversion material. As the injection current was increased, the EQE of the hybrid LEDs became progressively lower than that of the InGaN-based blue-violet LEDs. Temperature-dependent PL measurements showed that the emission properties of the CsPbBr_3_ QDs significantly deteriorated under ambient temperature. In particular, the activation energy for the temperature-dependent decrease in the EL intensity of the hybrid LEDs with green QDs was similar to that of the temperature-dependent PL intensity reduction in the green QDs. This indicates that the optical degradation of the hybrid LEDs can be ascribed to the thermal degradation of the green QDs under the high-injection-current region. To suppress the thermal degradation of the green QDs in hybrid LEDs, pulse injection can be applied to increase the emission efficiency of the hybrid LEDs with green QDs. Under the pulse injection conditions, the light-conversion efficiency of the green QDs was 22% that of the blue-violet InGaN-based LEDs under 100 mA, resulting in multicolor emissions from blue-violet to cyan.

## Data Availability

Not applicable.

## Supplementary Materials

The following supporting information can be downloaded at: https://www.mdpi.com/article/10.3390/ma16031290/s1, Figure S1: Schematic diagrams of CsPbBr_3_ QD on glass, InGaN-based LED and hybrid LED with CsPbBr_3_ QDs, Figure S2: The junction temperature measurements of hybrid LED using forward voltage method. Figure S3: Green emission stability of CsPbBr_3_ perovskite QDs in hybrid LEDs.
