# Supplementary material for: Monolithic Multicolor Emissions of InGaN-Based Hybrid Light-Emitting Diodes Using CsPbBr3 Green Quantum Dots
Source: Materials (Basel). 2023 Feb 2;16(3):1290. doi: 10.3390/ma16031290 (PMC9921764; doi:10.3390/ma16031290)
Supplement: Supplementary file 1 [file materials-16-01290-s001.zip › materials-2172648-supplementary.pdf]

Supplementary Information

Monolithic Multicolor Emissions of InGaN-Based Hybrid Light-Emitting Diodes Using $CsPbBr_3$ Green Quantum Dots

Jae-Hyeok Oh [1], Seung-Beom Cho [2], Il-Kyu Park [2] and Sung-Nam Lee [1,3,*]

[1] Department of IT & Semiconductor Convergence Engineering, Tech University of Korea, Siheung 15073, Republic of Korea
[2] Department of Materials and Science Engineering, Seoul National University of Science & Technology, Seoul 01811, Republic of Korea
[3] Department of Nano & Semiconductor Engineering, Tech University of Korea, Siheung 15073, Republic of Korea
* Correspondence: snlee@tukorea.ac.kr; Tel.: +82-31-8041-0721

S1. Schematic diagrams of $CsPbBr_3$ QD on glass, InGaN-based LED and hybrid LED with $CsPbBr_3$ QDs

S2. The junction temperature measurements of hybrid LED using forward voltage method

S3. Green emission stability of $CsPbBr_3$ perovskite QDs in hybrid LEDs

S1. Schematic diagrams of $CsPbBr_3$ QD on glass, InGaN-based LED and hybrid LED with $CsPbBr_3$ QDs

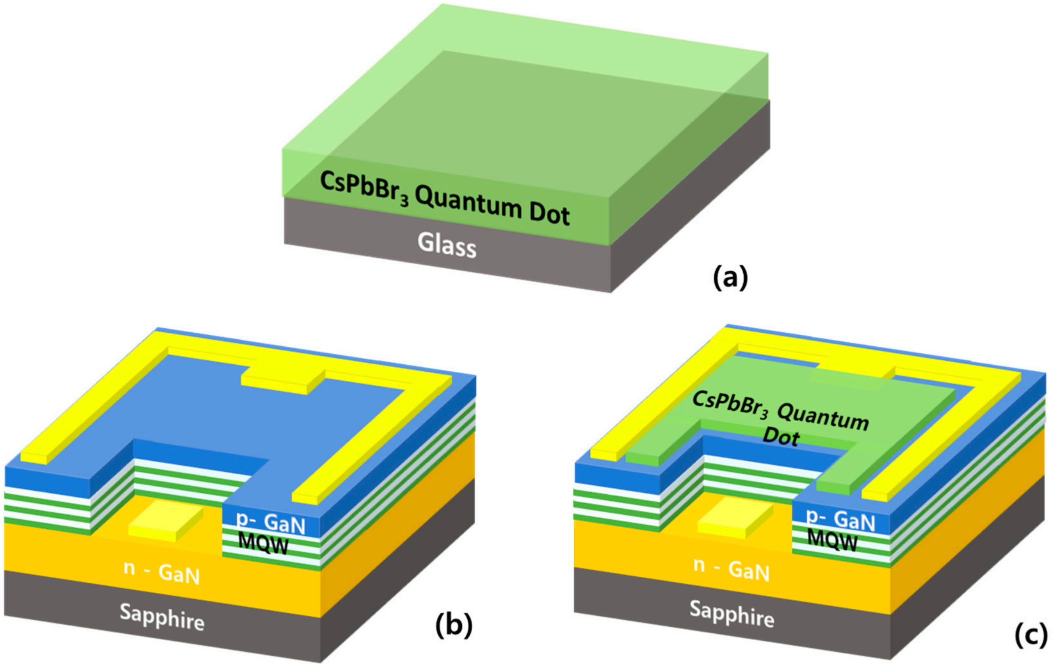

Figure S1. Schematic diagrams of $CsPbBr_3$ QDs on glass, conventional InGaN-based violet LED and hybrid LED with $CsPbBr_3$ QDs.

Figure S1(a) shows that $CsPbBr_3$ QDs were deposited on the glass substrate. After growing the InGaN-based violet LED structure, LED chips with lateral electrode structures were fabricated using a conventional mesa LED process with the Ti/Al n-electrode and Ni/Au p-electrode shown in Fig. S1(b). After an additional photolithographic process, $CsPbBr_3$ QDs were deposited on InGaN-based violet LEDs using a spin-coating process as shown in Fig. S1(c). For the spin-coating method, 50 µL $CsPbBr_3$ was added at 2000 rpm for 60 s, and then the n-hexane solvent was removed at 70 °C for 20 min in a nitrogen atmosphere.

S2. The junction temperature measurements of hybrid LED using forward voltage method

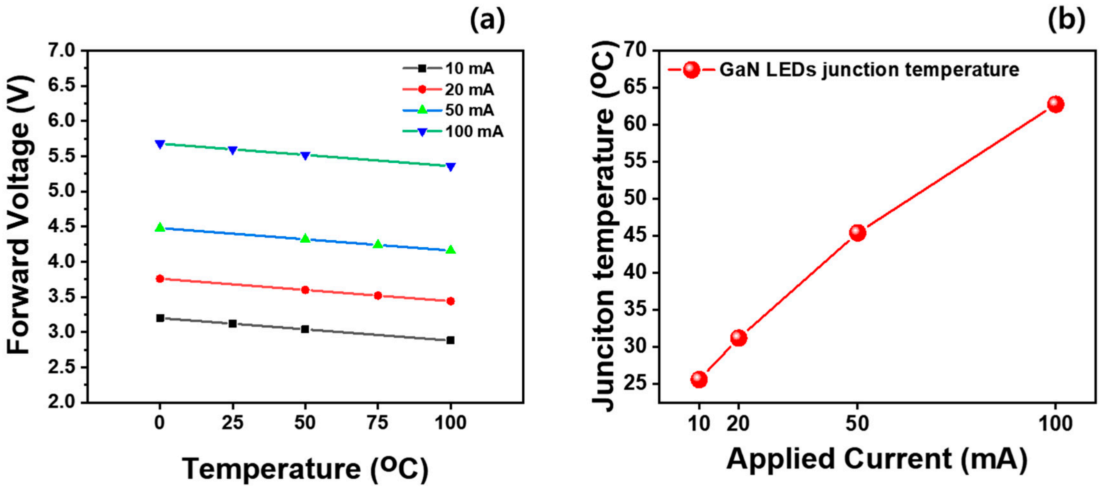

Figure S2. (a) Pulse calibration measurements of hybrid LED on the heating plate using a 1.0 μs pulse width and a 0.1% duty cycle and (b) the junction temperature of hybrid LED as a function of applied current.

The junction temperature of the LED was measured using the forward voltage method using the pulse calibration measurements to eliminate the heat generation. The pulse calibration measurements of the hybrid LED were performed on the temperature-controlled hot chuck from 25 to 100 °C. The relation ($V_f = aT_{heating\ plate} + b$) between forward voltage and junction temperature for each forward current can be obtained as shown in Fig. S1(a). Subsequently, the operation voltages of hybrid LED were measured by DC forward voltage. The junction temperature of hybrid LED can be achieved by the calibration measurement data for different injection currents as shown in Fig. S1(b). It indicates that as the injection current increased from 10 to 100 mA, the junction temperature of hybrid LED increased from 26.2 to 65 °C, respectively.

S3. Green emission stability of $CsPbBr_3$ perovskite QDs in hybrid LEDs

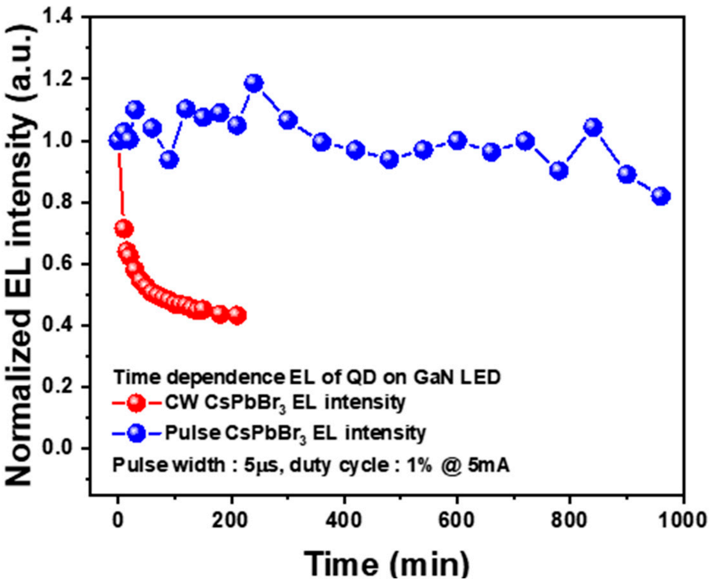

Figure S3. Normalized EL intensity of green emission of hybrid LEDs with CsPbBr3 QDs using cw and pulse operation (5.0 μs pulse width and 0.1% duty cycle) conditions.

The operation stability of the hybrid LEDs was evaluated by measuring the green emission intensity of the EL spectrum under cw and pulse operation conditions. Under the cw operation condition, the violet emission of the hybrid LED shows constant regardless of operation time, whereas the green emission of hybrid LED is significantly decreased to less than 50 % at an operating time of 100 min. However, in the pulse operation condition, the green emission of hybrid LEDs remained 82 % of its initial intensity up to 1000 min of operation time. It indicates that the green emission stability of $CsPbBr_3$ QDs in hybrid LEDs does not have high values of the commercially available level, but pulse operation condition can achieve superior green emission stability of hybrid LEDs compared to cw operation condition.
